# Novel method for detecting complement C3 deposition on *Staphylococcus aureus*

**DOI:** 10.1038/s41598-022-20098-7

**Published:** 2022-09-21

**Authors:** Toska Wonfor, Shuxian Li, Rhys W. Dunphy, Alex Macpherson, Jean van den Elsen, Maisem Laabei

**Affiliations:** 1 Department of Life Sciences, University of Bath, Bath, UK; 2 UCB Biopharma UK, Slough, UK

**Keywords:** Biological techniques, Immunology, Microbiology

## Abstract

The primary host response to *Staphylococcus aureus* infection occurs via complement. Complement is an elegant evolutionarily conserved system, playing essential roles in early defences by working in concert with immune cells to survey, label and destroy microbial intruders and coordinate inflammation. Currently the exact mechanisms employed by *S. aureus* to manipulate and evade complement is not clear and is hindered by the lack of accurate molecular tools that can report on complement deposition on the bacterial surface. Current gold-standard detection methods employ labelled complement-specific antibodies and flow cytometry to determine complement deposited on bacteria. These methods are restricted by virtue of the expression of the *S. aureus* immunoglobulin binding proteins, Protein A and Sbi. In this study we describe the use of a novel antibody-independent C3 probe derived from the staphylococcal Sbi protein, specifically Sbi-IV domain. Here we show that biotin-labelled Sbi-IV interacts specifically with deposited C3 products on the staphylococcal surface and thus can be used to measure complement fixation on wild-type cells expressing a full repertoire of immune evasion proteins. Lastly, our data indicates that genetically diverse *S. aureus* strains restrict complement to different degrees suggesting that complement evasion is a variable virulence trait among *S. aureus* isolates.

## Introduction

*Staphylococcus aureus* is an important human pathogen responsible for significant amount morbidity and mortality globally. *S. aureus* exists as a human pathobiont that intermittently or permanently colonises the anterior nares of approximately 30% of the human population^1^. Colonisation increases the risk of staphylococcal infection which can range from mild skin infections to life-threatening bacteraemia^2^. This pathogen gained notoriety as methicillin- resistant *S. aureus* (MRSA) which is now synonymous with multidrug-resistant *S. aureus* as clinical isolates are increasingly becoming resistant to the most common clinically used antibiotics. Worryingly, recent global epidemiological data has identified MRSA as one of the leading pathogens for deaths associated with antimicrobial resistance^3^.

All successful pathogens have evolved mechanisms to resist complement which are intimately aligned with their pathogenicity^4^. Complement is an essential component of innate immunity, playing a central role in defence against infection. Complement consists of more than 50 soluble and immobilized proteins, receptors, and regulators^5^. Microbial activation of complement occurs through a well-defined, tightly regulated series of enzymatic reactions originating from 3 different pathways, converging at the level of C3 convertase formation. C3 convertases instigate the cleavage of the central C3 molecule into the inflammatory modulator C3a and major opsonin C3b/iC3b, both prerequisites for efficient phagocytosis and microbial eradication. Amplification of C3b deposition occurs via the alternative pathway C3 convertase which also promotes the formation of C5 convertases directing the cleavage of C5 into C5a and C5b. C5a is a potent anaphylatoxin whereas C5b deposits onto cell membranes and initiates the formation of the membrane attack complex (MAC), resulting in lysis of susceptible cells^5,6^.

**Figure 1:**
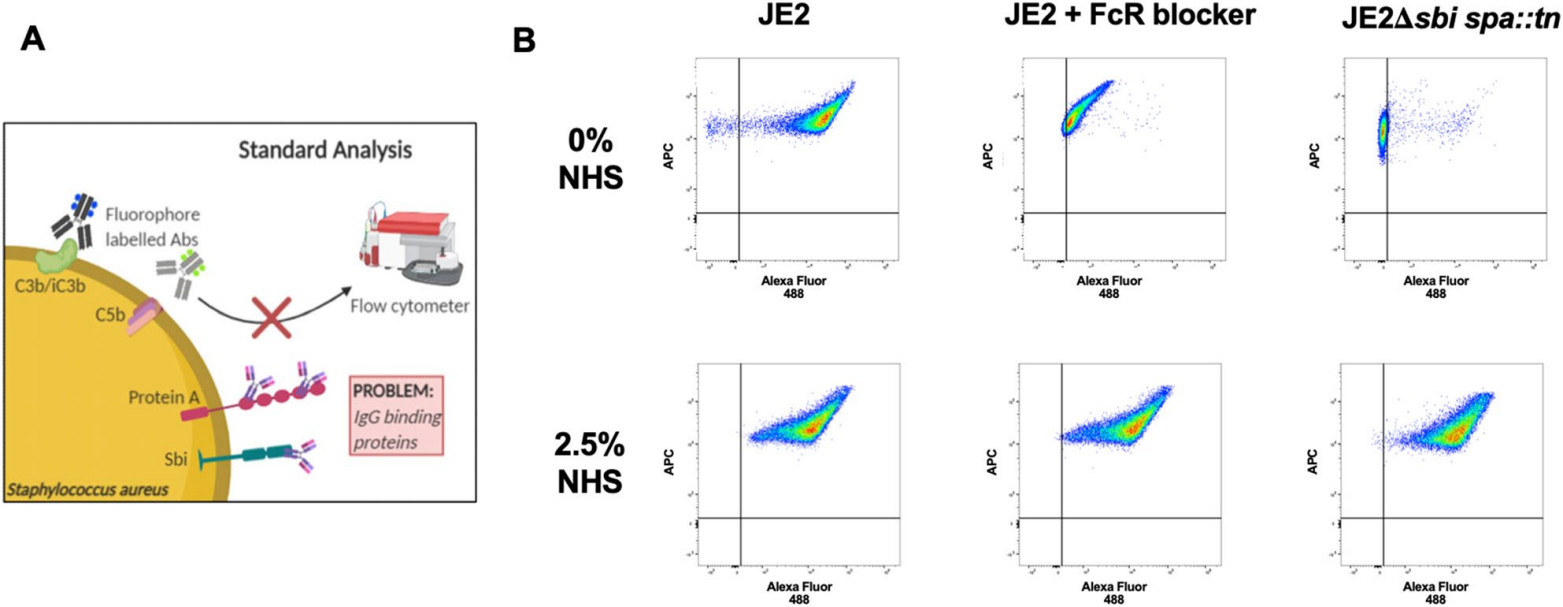
Non-specific binding of antibodies by *S. aureus* disrupts complement deposition detection by flow cytometry. (**A**) Schematic of Spa and Sbi interference with flow cytometry, figure created with permission using https://biorender.com/. (**B**) JE2 and JE2Δ*sbi spa::Tn* were grown to exponential phase, incubating with either normal human serum (2.5%) diluted in GVB^++^ buffer or buffer alone (0%). In addition, JE2 was incubated in 0% and 2.5% NHS in the presence of a commercial FcR blocking reagent (1:5). Opsonised bacteria were probed with polyclonal rabbit anti-human C3d and goat anti-rabbit AF488 and geometric mean fluorescence intensity (gMFI) was measured using a BD FACS Canto flow cytometer.

*S. aureus* possesses an extensive anti-complement arsenal^7^. Measuring the level of C3 deposition on the bacterial surface provides an insight into the strategies employed by bacteria to prevent complement activation. However, understanding the exact role of virulence factors in mediating complement evasion by evaluating C3 deposition on the bacteria surface has been hindered by the lack of appropriate phenotypic assays. Currently, accurate analyses of complement deposition on bacteria routinely use labelled complement-specific antibodies (Ab), which are detected and analysed using flow cytometry (Fig. 1A). This represents a significant problem in *S. aureus* as > 95% of clinical isolates express two Ab-binding proteins, protein A (Spa) and staphylococcal immunoglobulin-binding protein (Sbi), resulting in non-specific binding of Abs. Current methods to circumvent this issue use △*spa*△*sbi* double mutants. This is problematic as both molecules are important virulence factors that interfere with complement activity^8,9^. Therefore, use of deletion mutants to understand the relative contribution of individual complement evasins or to measure efficacy of immunotherapeutics can result in inaccurate predictions. Furthermore, reliance on double mutants prevents the determination of complement resistance in clinical isolates, restricting analysis to a limited number of laboratory strains. Recent methods have described using FcR blocking reagents to prevent non-specific binding of antibodies by Spa and Sbi with success, however differences in the ability of FcR blockers to completely eliminate non-specific binding of antibodies raised from different species has been reported^10^.

Sbi is a key immune evasion protein expressed by most *S. aureus* strains^11^. Sbi contains four N-terminal globular functional domains which interact with antibodies and complement. Sbi I-II consists of two immunoglobulin binding domains which are exposed on the staphylococcal surface and facilitate interaction with the Fc region of IgG^12^. Sbi III-IV domains bind the central complement protein C3 and remain buried when associated with the bacterial surface only becoming biologically active when Sbi is released^12,13^. The structure of Sbi-IV in complex with ligand C3d has been resolved identifying key residues located on helix α2 of Sbi-IV and the concave surface of C3d^14^. In this study we report the use of biotin labelled Sbi-IV as an accurate probe for C3 deposition on the surface of *S. aureus*, effectively eliminating detection-interference caused by *S. aureus* IgG binding proteins.

## Materials and methods

### Bacterial strains and growth conditions

*S. aureus* strains were routinely grown in tryptic soy broth (TSB), *Escherichia coli* DC10B and BL21(DE3) were grown in Luria Bertani (LB) medium at 37 °C with shaking at 180 rpm. *Lactococcus lactis* strain MG1363 was grown in M17 broth with 1% glucose (M17-G) at 30 °C without shaking. For *S. aureus* strain JE2∆*sbi spa*::Tn, erythromycin (5 μg/ml) was added to the growth medium. The presence of known complement evasin genes in genome-sequenced *S. aureus* strains were identified using AureoWiki^15^.

### Construction of the JE2∆*sbi spa*::Tn mutant

To construct the Spa/Sbi double mutant (JE2∆*sbi spa*::Tn) the *sbi* gene was deleted in the JE2 *spa::Tn* background sourced from the Nebraska transposon mutant library using the allelic exchange vector pIMAY*^16,17^. To create pIMAY*::*∆sbi*, 750 bp upstream and 750 bp downstream of *sbi* gene was synthesized as a single fragment and cloned into vector pUC18 by GenScript (pUC18::*∆sbi*). Both pIMAY* and pUC18::∆*sbi* were digested with enzymes XhoI and XmaI (NEB) and the *∆sbi* sequence was ligated into pIMAY*. pIMAY*∆*sbi* was transformed into *E. coli* DC10B before transforming into JE2 *spa::Tn* as described^18^. Allelic exchange was performed as described^17^ and deletion of *sbi* gene was confirmed by sequencing (Eurofins) with primers *sbi* OUT FW (GCTGTTCCAATGCTGACTAAAC) and *sbi* OUT RV (CTGGACCAGTTGGGTCTTGTG).

### Expression and purification of recombinant Sbi-IV

The pQE30-Sbi-IV plasmid^13^ was transformed into BL21(DE3) using heat shock and selection on LB agar with ampicillin (100 µg/ml). Sbi-IV expression was induced at OD_600nm_ 0.6 with 0.5 mM IPTG and grown for a further 3 h. The cells were harvested by centrifugation at 4000 × *g* at 4 °C and pellet resuspended in His A Buffer (50 mM Tris, 150 mM NaCl, 20 mM Imidazole, pH 7.4). To lyse the pellet, 300 µl of Protease Inhibitor Cocktail Set VII (Millipore Corp) was added before sonicating on ice in 10 s bursts for 10 min. The lysate was centrifuged for 30 min at 60,000 × *g* at 4 °C, and the supernatant filtered through a 0.45 µm filter before loading onto AKTA purifier (Amersham Pharmacia Biotech) with a 1 ml His-Trap HP column (Cytvia)*.* The bound protein was eluted with His B buffer (50 mM Tris, 150 mM NaCl, 500 mM Imidazole, pH 7.4.) Fractions containing the protein were identified using SDS-PAGE, pooled, and concentrated to 5 ml with VIVASPIN 500 (5 K MWCO) column. To remove remaining contaminants, purification was repeated using Size Exclusion Column HiLoad 16/600 Superdex 200 prep grade (Cytvia) and eluted with SEC buffer (20 mM Tris, 150 mM NaCl, pH 7.4). Fractions containing Sbi-IV were identified using SDS-PAGE, pooled, concentrated, and stored at − 80 °C. The protein concentration was determined using Pierce BCA Protein Assay Kit, following manufactures protocol (Thermo Fisher).

Sbi-IV R231A was generated using the Q5 Site-Directed Mutagenesis kit (NEB) following manufacturer’s instructions. Briefly, the pQE30-Sbi-IV plasmid was amplified using the included Q5 Hot Start HF polymerase with mutagenic primers: Mut.FW (TGAAAACAGAGCTTTAGCACAACGTGAAGTTAAC), and Mut.RV (ATTGAATCTTTTTCATTTAATTTTGAG). The PCR product was briefly incubated with KLD enzyme mix, then transformed into the NEB 5-alpha competent *E. coli*. The R231A mutation was confirmed via sequencing (Eurofins) with primer ‘pQE30 seq’ CAGGGTTATTGTCTCATGAGC. Protein expression and purification was performed as outlined above.

### Protein biotinylation

Biotinylation of Sbi-IV was performed using EZ-Link Sulfo-NHS-Biotinylation Kit (Thermofisher) following the manufactures protocol. Briefly, SEC buffer was exchanged to PBS (Dulbecco A, Oxoid) using the included Zeba Spin Desalting Column. Following this exchange, 283.5 µl of 10 mM Sulfo-NHS-LC Biotin was added to the protein solution and incubated on ice for 2 h. Excess biotin was removed using Zeba Spin Desalting column, and the final product aliquoted and stored at − 80 °C. For confirmation, labelled and unlabelled Sbi-IV were separated using a 4–20% SDS-PAGE gel (Bio-Rad) with 2 × non-reducing sample buffer (62.5 mM Tris pH 6.8, 4% SDS, 10% Glycerol, 0.01% Bromophenol Blue). The proteins were transferred to a 0.2 µm nitrocellulose membrane using Trans-Blot Turbo Transfer System (Bio-Rad). Membrane was blocked in 10% milk block (10% non-fat milk powder + 10 ml Tris Buffered Saline with Tween 20, pH 8.0—TBST Sigma) and placed on a rocker for 1 h at RT. Membrane was washed × 3 with TBST and probed with Ultra Streptavidin-HRP (Thermofisher) in 1:20000 dilution for 1 h. Proteins were detected using the ECL Detection Reagent (Amersham) and viewed using an Azure Biosystem 400 (Azure).

### Complement deposition assay

Overnight cultures of *S. aureus* were diluted 1:200 in fresh TSB and grown to mid-late exponential phase (OD_600nm_ = 0.5–0.6). Cells were centrifuged at 14,000 × *g* for 5 min and the pellet resuspended in 1 ml PBS. This wash was then repeated before normalising to OD_600nm_ 2. *S. aureus* cells were stained with 2 µM Cell Trace Far Red (Thermofisher) and incubated shaking at 37 °C for 20 min. Excess stain was removed by washing cells with 1% BSA/PBS before finally resuspending in GVB^++^ buffer (5 mM veronal buffer [pH 7.3], 0.1% [w/v] gelatine, 140 mM NaCl, 1 mM MgCl2, and 0.15 mM CaCl_2_). For *L. lactis,* an overnight culture was grown in 5 ml M17-G at 30 °C without shaking. One ml of overnight culture was harvested as described above but normalised to OD_600nm_ 1 in PBS. *L. lactis* was stained with 1 µM Cell Trace Far Red as described before and resuspended in GVB+ + buffer.

Normal human serum (NHS) was prepared from freshly drawn blood obtained from 8 healthy volunteers using BD vacutainer Serum CAT tubes. Blood was allowed to clot for up to 30 min at room temperature followed by incubation on ice for 1 h. Serum was collected following two rounds of centrifugation at 700 × *g* at 4 °C for 8 min. Serum fractions from individual donors were pooled, aliquoted and immediately stored at − 80 °C. All healthy volunteers provided written informed consent and all methods and experimental protocols were carried out in accordance with the recommendations of the University of Bath, Research Ethics Approval Committee for Health. The present study was approved by the University of Bath, Research Ethics Approval Committee for Health [reference: EP 18/19 108]. Heat-inactive serum (HIS) was prepared by heating serum at 56 °C for 30 min, compstatin-treated serum (CP40-HS) was prepared by incubating serum with CP40 (50 µM); for 20 min on ice before use; C3 depleted serum was obtained from CompTech (A314). Serum was diluted to indicated percentages in GVB^++^ buffer.

V-bottom 96-well plates were used for complement deposition assays. To enhance bacteria retention, V-bottom plates were incubated with 10% fetal calf serum/PBS for 30 min at room temperature and washed three times with 200 µl PBS. Stained bacteria and serum were mixed 1:1 in 96-well V-bottom plates for 30 min at 37 °C. Plate was centrifuged at 3,000 × *g* for 7 min (5810 R Eppendorf) and the supernatant discarded. If required, FcR blocking reagent (Miltenyi Biotec) was used prior to antibody probing at a concentration of 1:5 or 2:5 in 1% BSA/PBS with blocking occurring at 4 °C for 20 min. No difference in non-specific binding was observed between the two dilutions. Bacterial pellets were washed with 200 µl 1% BSA/PBS before resuspending with 100 µl of the primary probe either polyclonal rabbit anti-human C3d (Dako; 1:1000) or labelled Sbi-IV (0.175 μM) in 1% BSA/PBS. The plate was incubated at room temperature for 45 min, then washed again with 200 µl 1% BSA/PBS. The pellet was resuspended with 100 µl secondary probe either goat anti-rabbit IgG with Alexa Flour Plus 488 (Invitrogen) or Streptavidin Alexa Flour 488 (Invitrogen) diluted 1:1000 in 1% BSA/PBS. The cells were washed for a final time, before resuspending in 100 µl PBS and analysed on FACS CANTO (BD) using wavelengths 488 nm and 633 nm. Stained and unstained bacteria were used for accurate gating of bacteria and a minimum of 20,000 events were examined. The collected data was analysed using FlowJo™ v10 software (BD Life Sciences) www.flowjo.com.

### Statistical analysis

A one-way ANOVA or an unpaired T test was used to examine the statistical significance between experimental results (GraphPad Prism v9, GraphPad Software, San Diego, California USA, www.graphpad.com) where a *p* value of < 0.05 was considered to be statistically significant.

## Results

### Non-specific binding of antibodies by *S. aureus* interferes with complement deposition detection by flow cytometry

Flow cytometry is a gold-standard method for analysing complement deposition on microbial surfaces. *S. aureus* expresses two key immune evasion proteins, Protein A and Sbi, that bind to the Fc region of a suite of human and mammalian IgG and IgM antibodies, preventing the use of fluorophore labelled anti-complement antibodies to detect complement deposition (Fig. 1A). To demonstrate this we performed flow cytometry with *S. aureus* strain JE2 (USA300, clonal complex 8) and JE2 lacking immunoglobulin binding proteins, SpA and Sbi (JE2*Δsbi spa::tn*). These strains were incubated with and without normal human serum (NHS) and probed using rabbit-anti human C3d and goat-anti rabbit-AF488. In the absence of serum and C3 deposition, wild-type JE2 displays apparent C3 deposition due to recruitment of primary and secondary antibodies. This non-specific binding is lost in the double mutant when incubated in the absence of serum, whereas true deposition is observed following incubation in serum (Fig. 1B). We also investigated the use of a FcR blocking reagent as a preventative step to eliminate non-specific binding of rabbit-anti human C3d. FcR blocking reagent worked well to prevent non-specific binding of antibodies (JE2 0% NHS gMFI = 11,000 ± 341, reducing binding by 95%, however there was a fivefold increase in binding of antibodies between JE2 incubated with FcR blocker (gMFI = 551 ± 72) and the JE2 JE2*Δsbi spa::tn* mutant (gMFI = 98 ± 22). These data highlight the need for an alternative method for detecting C3 deposition on the staphylococcal surface.

### Labelled Sbi-IV reports complement deposition on non-pathogenic *L. lactis*

**Figure 2:**
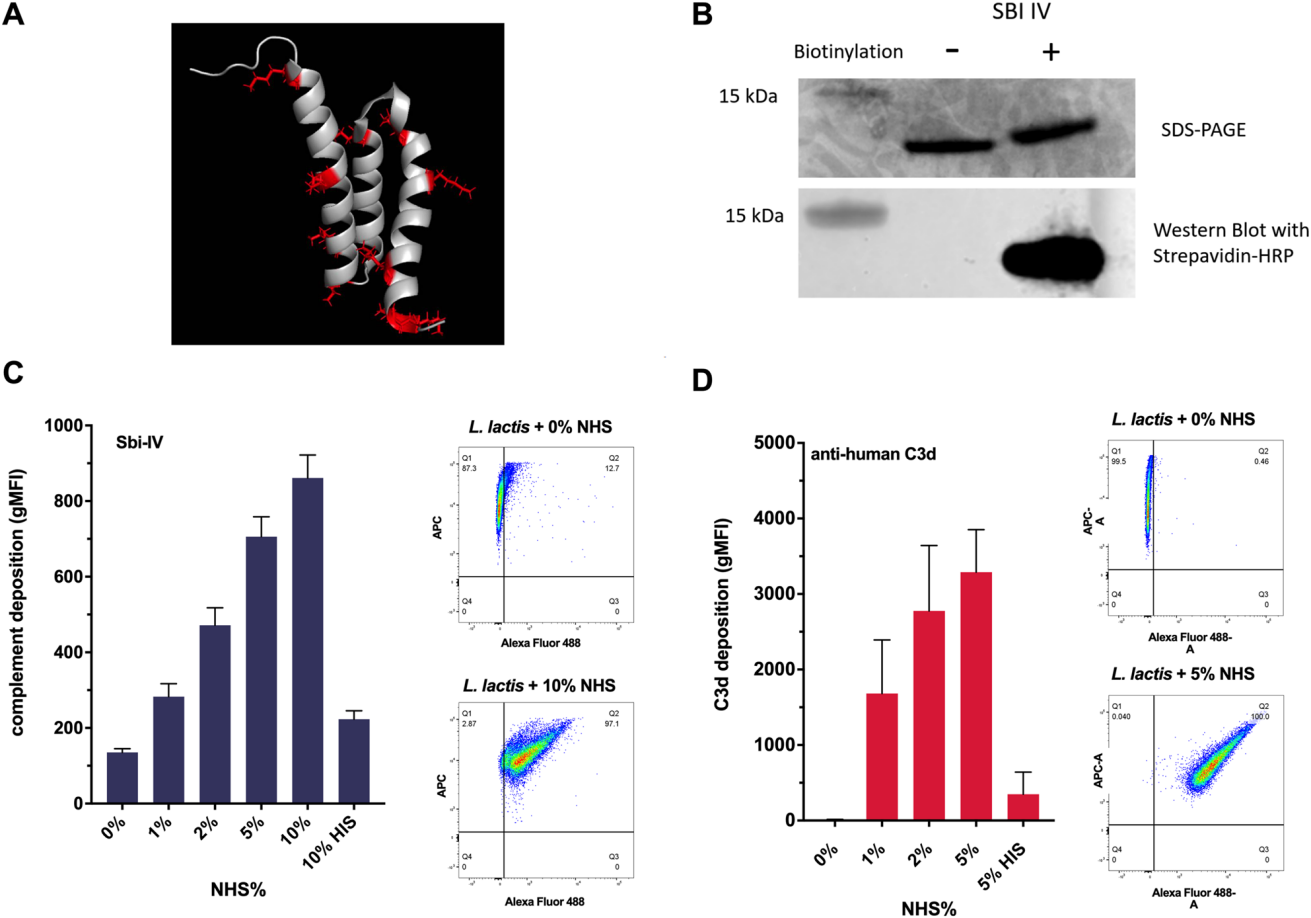
Purification and biotinylation of Sbi-IV and detection of complement deposition on non-pathogenic *L. lactis*. (**A**) *Ribbon* representation of Sbi-IV with lysines coloured in red (Protein Data Bank accession code 2JVH; figure prepared using PyMOL^29^. (**B**) SDS-PAGE and western blot analysis of biotinylated and non-biotinylated Sbi-IV. The SDS-PAGE gel has been stained with Coomassie, and the western blot has been probed with Streptavidin-HRP. (**C**,**D**) *L. lactis* has been incubated with increasing concentrations of normal human serum diluted in GVB^++^ buffer. (**C**) Geometric mean fluorescence intensity (gMFI) of complement deposition using biotinylated SBI-IV probe and (**D**) polyclonal rabbit anti-human C3d measured using a BD FACS Canto flow cytometer.

We investigated whether the complement C3 binding domain IV of the staphylococcal Sbi protein (Sbi-IV) could be used to detect C3 deposition on a microbial surface. Sbi-IV is an 11 kDa protein composed of three alpha helices containing 10 lysines which are amenable for biotinylation (Fig. 2A–B; Supp Fig. 1). To determine efficacy of our probe we analysed C3 deposition upon non-pathogenic *L. lactis* with both an anti-C3d antibody and biotinylated Sbi-IV (Fig. 2C–D)*. L. lactis* has no identified complement evasion proteins to impede complement deposition, does not express IgG binding proteins and analogous to *S. aureus* is impervious to complement mediated lysis. Our results showed that both labelled Sbi-IV and a polyclonal mixture of anti-C3d antibodies exhibited increased binding with higher concentrations of serum indicating that Sbi-IV bound complement coated *L. lactis* in a dose-dependent fashion.

### Labelled Sbi-IV probe detects complement C3 deposition on the *S. aureus* surface

**Figure 3:**
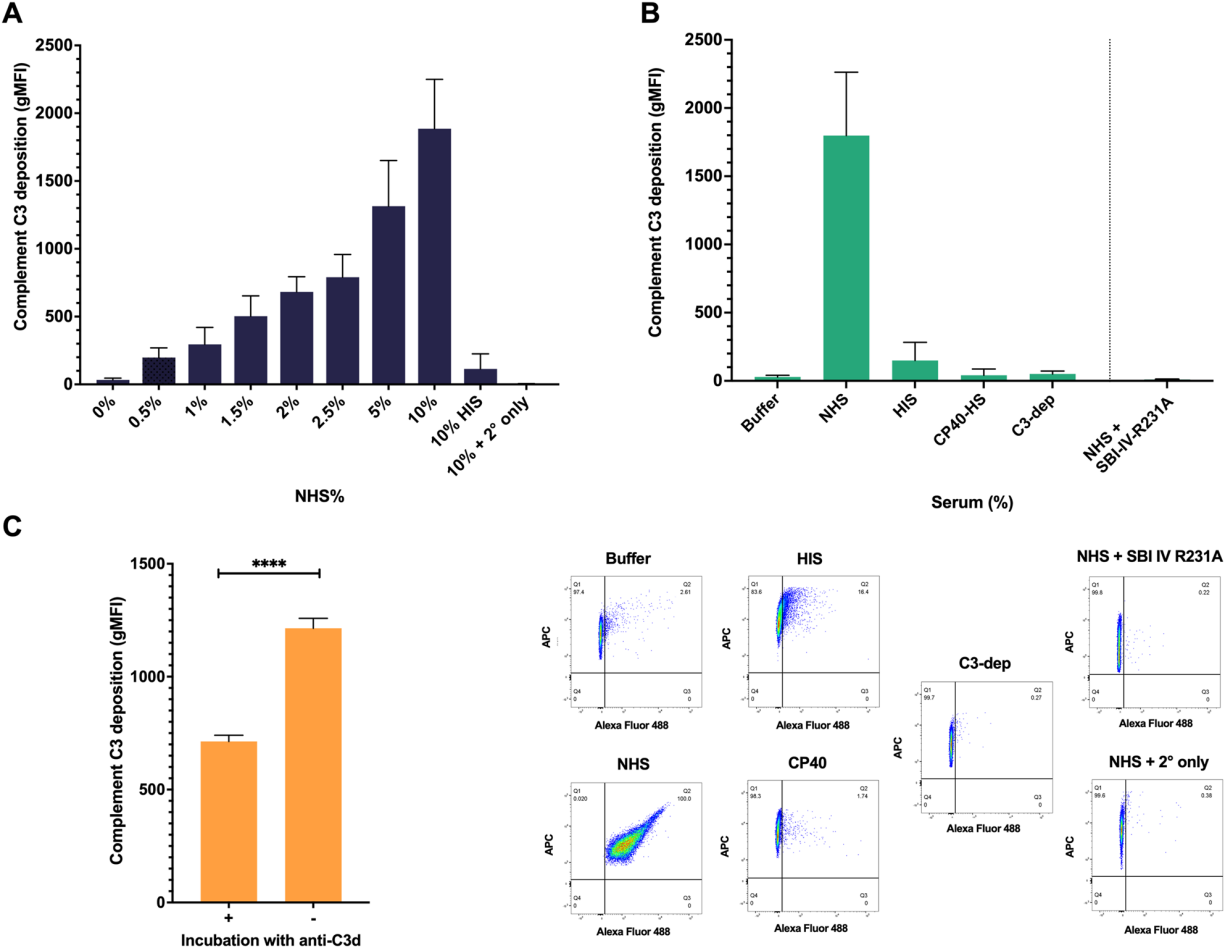
Labelled Sbi-IV detects complement C3 deposition on the *S. aureus* surface. (**A**) *S. aureus* strain JE2 was incubated with either increasing concentrations of normal human serum (NHS), 10% heat-inactivated human serum (HIS) or GVB^++^ buffer and probed with biotin-labelled Sbi-IV and streptavidin-AF488 with geometric mean fluorescence intensity (gMFI) measured using flow cytometry. (**B**) JE2 opsonised with either 10% NHS, 10% HIS; 10% C3-depleted serum (C3-dep) or 10% NHS treated with compstatin (CP40-HS; 50 µM). Complement C3 deposition detected using biotin-labelled Sbi-IV and streptavidin-AF488. JE2 was opsonised in 10% NHS and probed with biotin-labelled Sbi-IVR231A and streptavidin-AF488. Representative FACS scatter plots are shown for each of the conditions outline above. (**C**) JE2Δ*sbi spa::Tn* was opsonised in 10% NHS and incubated with polyclonal anti-human C3d antibody or not, washed and further probed with biotin-labelled Sbi-IV and streptavidin-AF488 with geometric mean fluorescence intensity (gMFI) measured using flow cytometry. Statistical differences were calculated (**C**) using an unpaired T-test. ****p < 0.0001.

Wild-type *S. aureus* strain JE2 was incubated in increasing concentrations of serum and probed with biotinylated Sbi-IV revealing a dose-dependent relationship between concentration of serum and bound Sbi-IV (Fig. 3A). To confirm that Sbi-IV binding was specific to C3 deposition we tested binding of Sbi-IV under a range of conditions (Fig. 3B). In the absence of serum and with 10% heat-inactivated serum we saw no significant Sbi-IV binding. Next, we incubated JE2 in 10% C3-depleted serum or 10% serum that was treated with the C3-specific peptide inhibitor compstatin (CP40), where in both cases Sbi-IV binding was abrogated. These results indicate that Sbi-IV interacts with C3 degradation products deposited on the staphylococcal surface. Furthermore, we generated a Sbi-IV R231A mutant which was previously shown to prevent Sbi-IV interaction with C3d^19^. Biotinylated Sbi-IVR231A was unable to detect deposited C3 on the *S. aureus* surface in the presence of 10% normal serum (Fig. 3B). Lastly, we examined whether labelling opsonised *S. aureus* (JE2*Δsbi spa::tn*) with antibodies against C3d (polyclonal anti-human C3d) prevented binding of labelled Sbi-IV. Our data shows a significant reduction in Sbi-IV binding in the presence of anti-human C3d antibodies (*p* < 0.0001). Combined our data indicates the specificity and utility of Sbi-IV as a probe for C3 deposition a bacterial surface.

### Variation in C3 complement evasion in *S. aureus*

**Figure 4:**
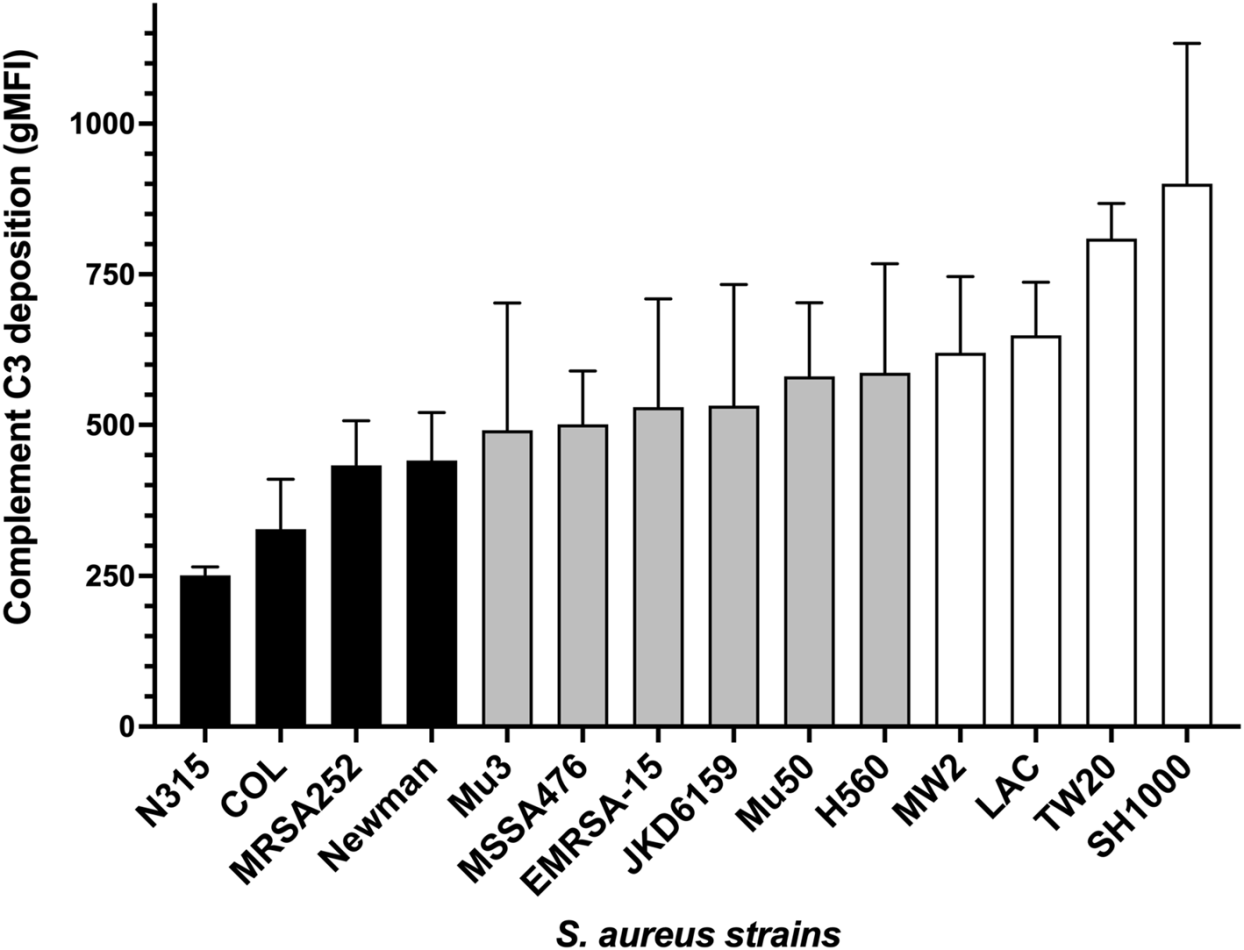
Variation in complement C3 deposition observed using a panel of genetically distinct MSSA and MRSA isolates. Each strain has been grown to mid-to-late exponential phase then normalised to OD_600nm_ 2 before incubating with 2.5% normal human serum. C3 deposition was observed by incubating biotin-labelled Sbi-IV and streptavidin-AF488. Geometric mean fluorescence intensity (gMFI) measured using flow cytometry. Statistical differences were calculated using a one-way ANOVA with Dunnett posttest.

To gain an insight of whether strain background impacts C3 deposition on *S. aureus*, we incubated a panel of 14 genome-sequenced, genetically diverse strains with pooled normal human serum (2.5%) and probed for C3 deposition with Sbi-IV (Fig. 4). The 2.5% serum concentration was chosen based on our initial titration experiments which shows a mid-range level of complement deposition on *S. aureus* (Fig. 3A). Complement opsonisation experiments indicate that strain N315 has significantly less C3 deposited than strains MW2 (*p* < 0.05), LAC (*p* < 0.05), TW20 (*p* < 0.001) and SH1000 (*p* < 0.0001). In addition, strains MRSA252, COL and Newman contain significantly less C3 deposition than TW20 (*p* < 0.05) and SH1000 (*p* < 0.001). Differences in C3 deposition between strains did not correlate with the absence of known complement evasin genes listed in Table 1, indicating that the observed significant differences in C3 deposition are not the result of an absence of key immune evasion gene(s) but likely the significant variation in expression of genes required for complement restriction.

**Table 1:** Presence of known complement evasin genes in *S. aureus* strains.

*Staphylococcus aureus* strains														
Gene	N315	COL	MRSA252	NWN	Mu 3	MSSA 76	E- 15	JKD	Mu 50	H560	MW2	LAC	TW 20	SH
*clfA*	+	+	+	+	+	+	+	+	+	+	+	+	+	+
*clfB*	+	+	+	+	+	+	+	+	+	+	+	+	+	+
*isdA*	+	+	+	+	+	+	+	+	+	+	+	+	+	+
*spa*	+	+	+	+	+	+	+	+	+	+	+	+	+	+
*sbi*	+	+	+	+	+	+	+	+	+	+	+	+	+	+
*cna*	+	+	+	+	+	+	+	+	+	+	+	+	+	−
*sdrE*	+	+	+	+	+	+	+	+	+	+	+	+	+	−
*cap*	+	+	+	+	+	+	+	+	+	+	+	*	+	+
*efb*	+	+	+	+	+	+	+	+	+	+	+	+	+	+
*ecb*	+	+	+	+	+	+	+	+	+	+	+	+	+	+
*sak*	+	−	+	+	+	+	+	+	+	+	+	+	+	+
*ssl7*	+	−	+	+	+	+	+	−	+	+	+	+	+	+
*sspB*	+	+	+	+	+	+	+	+	+	+	+	+	+	+
*sspA*	+	+	+	+	+	+	+	+	+	+	+	+	+	+
*aur*	+	+	+	+	+	+	+	+	+	+	+	+	+	+
*scpA*	+	+	+	+	+	+	+	+	+	+	+	+	+	+
*eap*	+	+	−	+	−	+	−	+	−	−	+	+	+	+
*scn*	+	−	+	+	+	+	+	−	+	+	+	+	+	+

NWN: Newman; E-15: EMRSA-15 (HO 5096 0412); JKD: JKD6159; LAC: USA300_FPR3757; SH1000: NCTC8325.

*Denotes mutations in capsular polysaccharide cap5 locus in USA300 (Boyle-Vavra et al. 2015 mBio).

## Discussion

Neutrophils are the major cellular component of innate immunity, representing an essential primary defence against staphylococcal infections^20^. Antibody and complement mediated opsonization of *S. aureus* directs efficient recognition by neutrophils resulting in *S. aureus* ingestion, intracellular processing triggering oxidative and non-oxidative mediated killing and effective elimination. Given the importance of complement in enhancing phagocytosis, understanding how *S. aureus* interferes with complement-mediated immune elimination may provide future targets for therapeutic intervention.

Several strategies have been used to by-pass interference from IgG binding proteins when examining complement deposition on the *S. aureus* surface. Routinely Spa/Sbi double deletion mutants have been used. Aside from the fact that both Spa and Sbi are important immune evasins impacting complement deposition, absence of Spa has been shown to impact expression of other virulence factors^21^. FcR blocking reagent has been used to reduce Spa/Sbi mediated non-specific binding^10^ and our data corroborate these observations. However we observed a fivefold difference in geometric mean values between JE2 incubated with Fc blocker and JE2*Δsbi spa::tn* mutant, indicating that FcR blocking did not eliminate all non-specific binding. Previous work using FcR blocking reagent also reported significant brand and species-dependent variability where authors have suggested that optimisation and validation of the use of FcR blockers is required. Moreover, genetic background of *S. aureus* strains influence the level of Spa expression^21^ which will impact on the utility of FcR blockers, where high level Spa expression will likely result in higher non-specific binding even in the presence of FcR blocker.

We sought to develop an antibody independent C3 probe to monitor complement C3 deposition on *S. aureus*. Previous surface plasmon resonance experiments conducted by our group have illustrated that the strongest binding interaction of purified Sbi III-IV and Sbi-IV was with C3 degradation products iC3b and C3d^11^. Gordan et al.have observed that C3 deposition and cleavage to iC3b occurs rapidly when *S. aureus* is opsonised in pooled human serum with C3b (17%), iC3b (64%) and C3d (19%) making up the C3 degradation products on the staphylococcal surface^22^. Taken together, these studies suggested that Sbi-IV could be used as a suitable probe to measure C3 deposition on *S. aureus* and thus could be used to determine complement opsonisation on clinical isolates of *S. aureus* expressing IgG binding proteins.

By using a non-pathogenic Gram-positive host, we showed labelled Sbi-IV detects complement deposition in a dose-dependent fashion, equivalent to detection by polyclonal anti-C3d antibodies (Fig. 2B–C). Next, we confirmed that Sbi-IV can report on complement deposition on wild-type *S. aureus* strain JE2 with increasing binding associated with increasing percentages of serum opsonisation. Importantly, significant non-specific staining of un-opsonised wild-type *S. aureus* was observed with the anti-C3d antibody, which was circumvented by staining with Sbi-IV. Previous data have indicated that Sbi can bind the fluid-phase complement regulators Factor H (FH) and Factor H related protein 1 (FHR-1) via domains III and IV via a tripartite complex with C3 deposition fragments^9,23^. Therefore, we investigated the specificity of Sbi-IV interaction with C3 degradation products deposited on the staphylococcal surface by flow cytometry. Compstatin is a peptidic C3-targeted inhibitor preventing the convertase-mediated cleavage of native C3 in the active fragments C3a and C3b, preventing C3b deposition and amplification of C3 cleavage^24^. We opsonised *S. aureus* in either C3-depleted serum or serum treated with compstatin and observed no binding of Sbi-IV indicating that Sbi-IV requires C3 degradation to be deposited for interaction. Next, we opsonised *S. aureus* in normal serum, incubated cells with polyclonal anti-C3d antibodies and examined Sbi-IV interaction (Fig. 3C). We observed a 50% reduction in Sbi-IV binding following incubation with polyclonal anti-C3d antibodies, confirming that C3d is a major epitope for Sbi-IV interaction. Lastly, we tested the ability of a previously described C3d binding mutant, Sbi-IVR231A^11^, to detect C3 deposition on *S. aureus.* This mutant showed no interaction with serum opsonised *S. aureus* (Fig. 3B). Combined our analysis shows that Sbi-IV binds specifically to C3 degradation products decorated on bacteria and is an appropriate C3 probe to specifically examine complement fixation on the staphylococcal surface.

*S. aureus* expresses a suite of virulence factors that are associated with pathogenicity^25^. Previous research from our group has shown that *S. aureus* virulence phenotypes such as cytotoxicity are highly variable both between and within MRSA lineages^26,27^. These studies underscore the complexity in *S. aureus* virulence regulation and suggests that there are undiscovered regulatory circuits that modulate virulence regulation. *S. aureus* complement evasion is an understudied area of the *S. aureus* virulence repertoire and consists of both secreted complement evasins and cell wall anchored proteins^7^. To gain an insight into the variability of *S. aureus* C3 evasion mechanisms we examined C3 deposition using 14 genetically distinct, genome-sequenced *S. aureus* isolates using labelled Sbi-IV. Our data indicates that, like previously measured virulence phenotypes, certain *S. aureus* isolates restrict C3 deposition to a higher degree with a greater than threefold difference observed between tested isolates. This data represents a snapshot at the level of variation in C3 deposition, suggesting distinct complement evasion strategies may be employed by *S. aureus*.

This Sbi-IV C3 deposition approach will be useful in screening larger cohorts of clinically relevant, genome-sequenced *S. aureus* isolates and in conjunction with genome-wides association studies^25^ has the potential to identify factors associated with enhanced immune evasion, providing a greater understanding of *S. aureus* pathogenicity.

Given the specificity of Sbi-IV to C3 activation products, we are currently investigating the potential of fluorescent or radio-labelled Sbi-IV to act as a biomarker of complement activation. Recently, non-invasive imaging using ^99m^Technetium-labelled recombinant complement receptor 2 has been used to detect C3d deposition in a murine model of myocardial ischaemia–reperfusion injury^28^. We envisage that labelled Sbi-IV may be used as a probe to detect tissue inflammation, compatible with non-invasive in vivo diagnostics or monitoring disease progression.

## Supplementary Information

Supplementary Information.

## Data Availability

The datasets used and analysed during the current study are available from the corresponding author on reasonable request.
