# Supplementary material for: Novel method for detecting complement C3 deposition on Staphylococcus aureus
Source: Sci Rep. 2022 Sep 21;12:15766. doi: 10.1038/s41598-022-20098-7 (PMC9492775; doi:10.1038/s41598-022-20098-7)
Supplement: Supplementary file 1 — Supplementary Information. [file 41598_2022_20098_MOESM1_ESM.pdf]

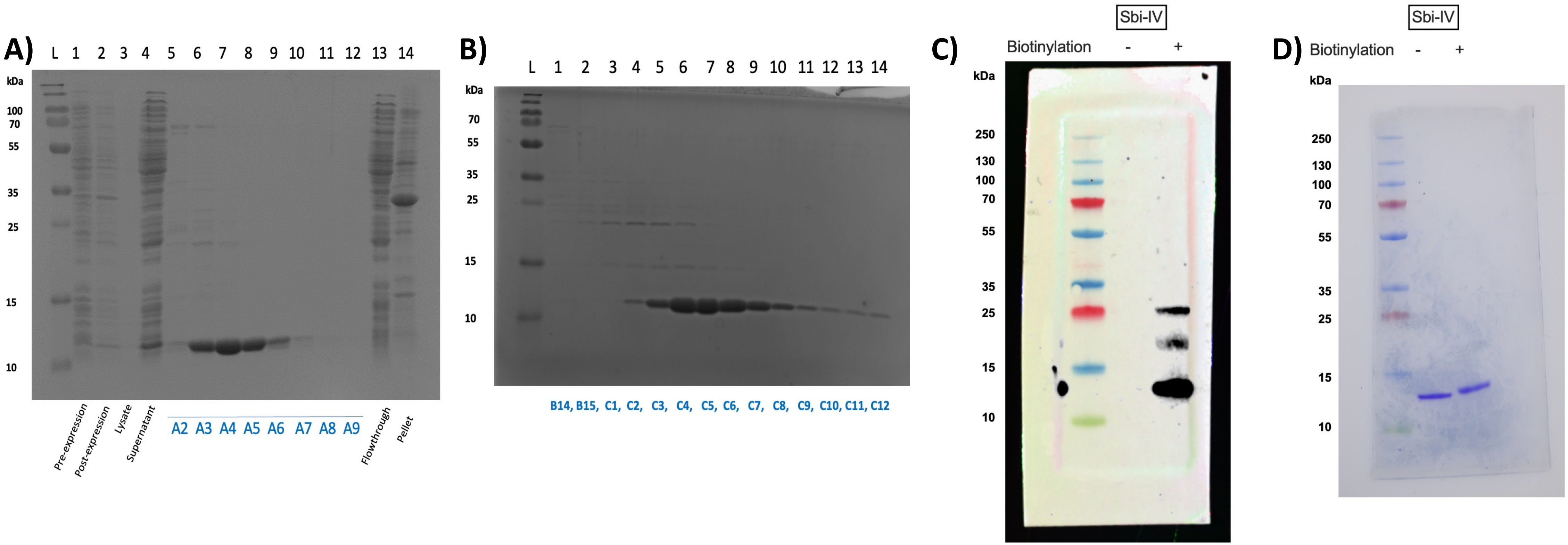

Supplementary Figure 1. A) SDS-PAGE gel of aliquots taken throughout His-Tag purification. A culture of *E. coli* pQE30-SbiIV was grown to OD 0.6 (lane 1). 0.5M IPTG was added to the culture and grown for a further 3 hrs (lane 2). The cells were lysed using sonication (lane 3) and the lysed culture was centrifuged. Supernatant from the culture was loaded onto an AKTA (lane 4) and passed through a His-Trap HP column. Non bound proteins were washed off the column (lane 13) and Sbi-IV was eluted with a high imidazole buffer, and collected in 2 ml aliquots (lane 5 to 12). Expected size of purified Sbi-IV is ~10kDa; aliquots A3 to A6 were pooled for further purification using size exclusion chromatography (SEC). **B)** SDS-PAGE gel of aliquots taken following SEC purification. Aliquots C4 to C12 were pooled together and concentrated. Ladder used was PageRuler plus (Thermofisher). **C)** Western Blot and **D)** SDS-PAGE analysis of biotinylated and non-biotinylated Sbi-IV. For SDS-PAGE 1 µg of each protein and has been stained with Coomassie, and the western blot uses 100 ng of each protein and has been probed with Streptavidin-HRP. Marker used is PageRuler Plus Protein Ladder (Thermofisher).
